# Molecular Characterization of Melanoma Cases in Denmark Suspected of Genetic Predisposition

**DOI:** 10.1371/journal.pone.0122662

**Published:** 2015-03-24

**Authors:** Karin A. W. Wadt, Lauren G. Aoude, Lotte Krogh, Lone Sunde, Anders Bojesen, Karen Grønskov, Nine Wartacz, Jakob Ek, Morten Tolstrup-Andersen, Mette Klarskov-Andersen, Åke Borg, Steffen Heegaard, Jens F. Kiilgaard, Thomas V. O. Hansen, Kerenaftali Klein, Göran Jönsson, Krzysztof T. Drzewiecki, Morten Dunø, Nicholas K. Hayward, Anne-Marie Gerdes

**Affiliations:** 1 Department of Clinical Genetics, University Hospital of Copenhagen, Copenhagen, Denmark; 2 QIMR Berghofer Medical Research Institute, Brisbane, Australia; 3 Department of Clinical Genetics, University hospital of Odense, Odense, Denmark; 4 Department of Clinical Genetics, University hospital of Århus, Århus, Denmark; 5 Department of Clinical Genetics, Vejle hospital, Lillebaelt Hospital, Vejle, Denmark; 6 Department of Cellular and Molecular Medicine, University of Copenhagen, Copenhagen, Denmark; 7 Department of Oncology, Lund University and Skåne University Hospital, Lund, Sweden; 8 Department of Ophthalmology, Glostrup Hospital, University of Copenhagen, Denmark; 9 Eye Pathology Institute, Department of Neuroscience and Pharmacology, University of Copenhagen, Copenhagen, Denmark; 10 Center for Genomic Medicine, Rigshospitalet, Copenhagen University hospital, Copenhagen, Denmark; 11 Department of Plastic Surgery, Breast Surgery and Burns, Rigshospitalet, Copenhagen University Hospital, Copenhagen, Denmark; University of Connecticut Health Center, UNITED STATES

## Abstract

Both environmental and host factors influence risk of cutaneous melanoma (CM), and worldwide, the incidence varies depending on constitutional determinants of skin type and pigmentation, latitude, and patterns of sun exposure. We performed genetic analysis of *CDKN2A*, *CDK4*, *BAP1*, *MC1R*, and *MITF*p.E318K in Danish high-risk melanoma cases and found *CDKN2A* germline mutations in 11.3% of CM families with three or more affected individuals, including four previously undescribed mutations. Rare mutations were also seen in *CDK4* and *BAP1*, while *MC1R* variants were common, occurring at more than twice the frequency compared to Danish controls. The *MITF* p.E318K variant similarly occurred at an approximately three-fold higher frequency in melanoma cases than controls. To conclude, we propose that mutation screening of *CDKN2A* and *CDK4* in Denmark should predominantly be performed in families with at least 3 cases of CM. In addition, we recommend that testing of *BAP1* should not be conducted routinely in CM families but should be reserved for families with CM and uveal melanoma, or mesothelioma.

## Introduction

Cutaneous melanoma (CM) accounts for 95% of melanoma cases and the incidence of CM in Denmark increased by 63.5% for males and 48.5% for females from 2003–2012 [1], making Denmark a high incidence melanoma country with age-standardized incidence rates of 32 and 35 per 100,000 for males and females, respectively. CM represents a significant public health burden, and was the most frequent type of cancer diagnosed in Danish women aged 15–30 years in 2012 [1]. Exposure to ultraviolet radiation (UVR) is the most well-established environmental risk factor for CM, but genetic components are also significant; an Australian twin study estimated that 55% of the variation in liability to CM is due to genetic effects [2]. A large Nordic epidemiologic study has shown that having a first-degree relative with CM is associated with a 2-fold increase in the risk of CM, rising to between 5-fold and 21-fold with multiple affected first-degree relatives [3]. Other known risk factors for CM are high nevus count, multiple atypical nevi, fair skin, red hair color, history of sunburn, use of indoor tanning, and previous melanoma [4–8].

Familial melanoma accounts for around 5–10% of CM cases and several high-risk genes have been identified. Mutations are most frequently seen in *CDKN2A*, where pathogenic mutations are detected in 20–40% of families with three or more cases of CM [9]. *CDKN2A* encodes two proteins through alternatively spliced transcripts, INK4A(p16) and ARF(p14). Both proteins affect cell cycle regulation; p16 inhibits the activity of CDK4 and CDK6, and thereby influences pRb regulated G1 to S-phase progression. The p14 protein affects the p53 pathway, which induces cell cycle arrest and apoptosis [10]. Other high-risk melanoma genes have been discovered: cyclin-dependent kinase 4 (*CDK4*) [11], BRCA-1 associated protein (*BAP1*) [12], and recently via exome sequencing of dense melanoma families, several new high-risk genes affecting telomere functions have been identified: *POT1*, *ACD*, *TERF2IP* and *TERT* [13–16]. However, mutations in these other high-risk genes are rare and each account for a minority of melanoma-dense families. In *CDK4* only two mutations (p.R24H, p.R24C), affecting binding to p16 [11], have been identified. Families with *CDK4* and *CDKN2A* mutations have similar phenotypes regarding CM, with cases frequently having multiple primary melanoma (MPM), early onset CM, and high numbers of clinically atypical nevi [17]. In a subset of families with *CDKN2A* mutations, an increased risk of pancreatic cancer has been reported. The precise relationship between mutations in *CDKN2A* and pancreatic cancer is unknown, but pancreatic cancer has predominantly been reported in Swedish, Italian, Dutch and North American CM families [9,18], and mainly with mutations affecting ankyrin repeats 3 and 4 [19].

Apart from high risk CM genes, two moderate risk genes are known, melanocortin receptor 1 (*MC1R*) and microphthalmia-associated transcription factor (*MITF*). *MC1R* is highly polymorphic in the Caucasian population and the variants most strongly associated with red hair color (designated R alleles) confer a per-allele risk of ∼2-fold for CM [20]. With the binding of α-melanocyte-stimulating hormone (α-MSH) to MC1R on melanocytes, synthesis of eumelanin is stimulated [21]. R alleles of *MC1R* lead to decreased or absent ability to activate the cAMP pathway upon binding of α-MSH, and inefficient stimulation of eumelanogenesis, resulting in a higher concentration of the red-yellow pheomelanin [22,23]. Eumelanin protects melanocytes from UVR damage, whereas pheomelanin is phototoxic by production of reactive oxygen species [24].

One mutation in *MITF* (p.E318K) is linked to moderate (∼2-fold) increased risk of CM and renal cell carcinoma (RCC). The mutation causes impaired sumoylation and altered regulation of several of the targets of MITF [25,26]. The p.E318K mutation is associated with non-blue eye color and increased nevus count. Additionally, population-based genome-wide association studies (GWAS) have located a number of low risk SNPs for CM, predominantly in genes related to melanogenesis, melanocyte differentiation, DNA repair, and immunological pathways [27–29].

In sharp contrast to CM, the incidence of uveal melanoma (UM) has been constant over the last 50 years, indicating little influence of lifestyle and patterns of sun exposure to the development of UM [30], and thus, possibly a stronger genetic basis. UM is the most common primary intraocular malignancy, with an annual incidence of approximately 2–8 per 1,000,000 [31]. The incidence is considerably lower in individuals with dark pigmentation. Several epidemiological studies have shown that predisposition in Caucasians is associated with light skin color, blond hair and blue eyes [32]. UM is located in the choroid, ciliary body, or iris, with only the latter potentially being exposed to solar ultraviolet light. To date, only one high penetrance UM predisposition gene (*BAP1*), has been identified. *BAP1* is a tumor suppressor gene and mutations have been identified in around 40 families with accumulation of UM, CM, mesothelioma, RCC, and basal cell carcinoma (BCC) [12,33–35]. There have been isolated reports of UM in *CDKN2A* mutation carriers [36,37], and *BRCA2* mutation carriers [38,39], but in light of the many families published with mutations in these two genes, and only single reports of UM, the risk of UM in carriers of *CDKN2A* or *BRCA2* mutations is probably low.

To date there has been no large study of genetic alterations in Danish high-risk melanoma cases, and we were intrigued by a clinical observation of an apparently low frequency of *CDKN2A* mutations when testing was conducted in a clinical genetic setting. A low frequency of *CDKN2A* mutations has previously been reported in German and Latvian studies [40,41]. Here, we examined the frequency of *CDKN2A*, *CDK4*, *BAP1*, *MC1R* and *MITF* (p.E318K) mutations in a large sample of Danish high-risk CM and UM cases.

## Material and Methods

### Ethics

The project was approved by the Danish data-protection agency and the ethics committee of the capitol region of Copenhagen (H-3-2011-050). All participants signed consent forms. The study has been conducted in accordance with the tenets of the Helsinki Declaration.

### Study population

**Fig 1:**
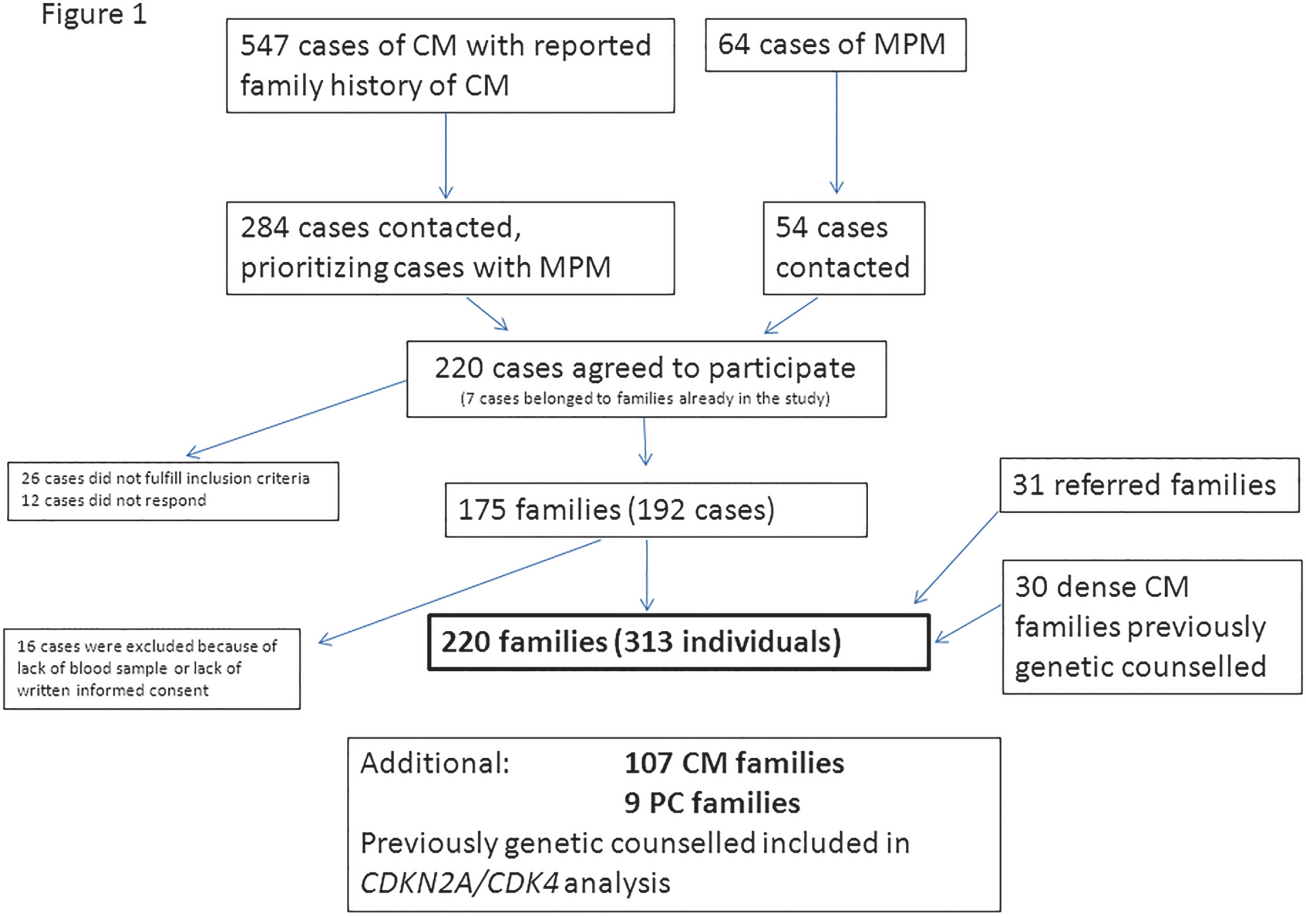
Flow-chart of melanoma cases included in the study.

The Danish melanoma registry is a nationwide registry established in 1985. The registry records detailed information about patients diagnosed with melanoma and also contains information about self-reported family history of melanoma. From the registry we retrieved 547 CM cases with reported family history of melanoma, and information on 64 individuals who developed 3 or more melanomas. We contacted 284 of the 547 individuals, prioritizing cases with MPM and also those who reported a family history of CM. We also contacted 54 of the 64 persons with MPM and no report of other melanoma cases in the family. We did not contact all of the isolated MPM cases, because many of these patients were fair skinned and frequent users of indoor tanning facilities, and were judged less likely for finding mutations in high-risk melanoma genes, than patients with a family report of melanoma. In total we contacted 338 individuals by letter in 2011–13, and 220 (65%) agreed by letter to participate in the study. Twenty-six persons did not fulfill the inclusion criteria (melanoma before 40 years or melanoma in a first degree relative or MPM) at first contact, and were excluded, and 12 persons did not respond to subsequent contact. Seven persons belonged to families already included in the study. We contacted all available individuals with CM in the families. In total 192 (57%) persons from 175 families participated. In addition, 31 individuals were referred to genetic counseling for suspicion of familial melanoma during the study period and were included. Thirty-four dense melanoma families, who previously had received genetic counseling, were contacted and 30 families agreed to participate in the study. Sixteen participants were excluded because they failed to have a blood sample drawn at the local hospital or they did not return the written informed consent. In total, 313 members of 220 families agreed to participate in the study (Fig. 1), and genetic analysis of *CDKN2A*, *CDK4*, *MC1R and MITF* was performed. *CDKN2A* and *CDK4* were examined in one melanoma case from each family, while *MC1R* and *MITF* were examined in all available CM and UM cases from the families. In families with a *CDKN2A* mutation, *MC1R* was examined in all mutation carriers.

An additional 107 families with CM and 9 families with pancreatic cancer had previously received genetic counseling and analysis of *CDKN2A* and *CDK4*. Information of cancer occurrence in these families was included in the analysis of *CDKN2A* and *CDK4* alterations.

*BAP1* was analysed in a subset of participants in the project (including all families with occurrence of UM, mesothelioma, or RCC) and in 12 individuals with isolated UM. In total, *BAP1* was analysed in 94 CM families, in 10 cases of CM < 40 years, in 23 sporadic cases of MPM, in 16 UM-CM families, and in 12 cases of UM.

### Samples

Blood samples were collected from participants and if possible from family members with CM. In families with a *CDKN2A* mutation, blood samples were also collected from healthy mutation carriers. DNA was extracted from whole blood using standard methods.

### Mutation analysis

#### Targeted next-generation sequencing (T-NGS)

164 families were screened for variants in *BAP1* and *CDKN2A* in a targeted sequencing approach using Ion AmpliSeq library kits (Life Technologies, CA, USA). Custom designed primer pools covering the two genes were designed to have mean coverage of 40X across the desired regions with amplicon lengths of 150 bp to 250 bp. 10 ng of genomic DNA from each proband were amplified. *BAP1* and *CDKN2A* had coverage of 96% and 97% respectively. Libraries were generated in half-volume amplification reactions. Ion Xpress Barcode adapters 1–64 were used to pool samples. The unamplified libraries were purified using Agencourt Ampure XP reagent (Beckman Coulter, CA, USA) in order to minimise fragments that were smaller than 100 bp and increase the proportion of on-target reads in downstream applications. Libraries were equalised to ∼100 pM using Ion Library Equalizer kits before being combined into a single sample. A Qubit 2.0 (Life Technologies, CA, USA) was used to assess the quality of the template enrichment before isolation of the template-positive ion sphere particles. The template was loaded onto Ion 318v2 chips and run on a Personal Genome Machine (Life Technologies, CA, USA) with 500 run flows per chip. The sequence data were analysed using Torrent Suite software and the output data filtered to minimise false positives. The criteria were: variants were required to have a minimum of 4 reads, the read count of the variant allele had to be a least 20% of the total read count, and quality score had to be greater than 40. Variants occurring in the NHLBI Exome Sequencing Project (ESP6500) with minor allele frequency (MAF) >0.01, and synonymous variants were excluded. Variants of interest were verified using Sanger sequencing.

#### High-resolution melting analysis

130 blood samples were screened with high-resolution melting analysis for *CDK4* mutations p.R24C or p.R24H [42]. Positive controls were included in every run. Primers and conditions are available upon request.

#### Sanger sequencing

Standard methods for Sanger sequencing were used to screen an additional 196 samples for mutations in *CDKN2A* and *CDK4;* 29 samples for mutations in *BAP1*; 280 samples for variants in *MC1R*, in which the following five variants: p.D84E, p.R151C, p.R160W, p.D294H, p.R142H; and null mutations, were classified as R variants and others were classified as r variants, except synonymous changes, which were counted as wild-type [20]. 296 samples were assessed for *MITF* p.E318K by a standard TaqMan assay.

The primers used are listed in Supplementary information.

### Statistical methods

Ordered logistic regression was used to calculate likelihood-ratios. Hazard ratios for *CDKN2A* mutation carriers were calculated using Cox regression. A Cox proportional-hazards model was used to generate the survival curve showing age-specific probability of melanoma development for *CDKN2A* mutations carriers.

## Results

### CDKN2A

Using T-NGS 131 samples had a mean sequencing coverage of 30X or above for *CDKN2A*, which was considered sufficient for accurate mutation screening.

13 of 327 cases with early onset CM or MPM carried mutations in *CDKN2A* (Table 1). Three mutations: c.47_50del p.(L16Pfs*9), c.62G>A p.(R21K), and c.94_99dup p.(L32_E33dup) mutations have not previously been described.

**Table 1:** Characteristics of the 13 individuals/families with identified *CDKN2A* mutations.

Location of mutation	INK4A Nucleotide change (NM_000077.4)	p16 Protein change (NP_000068.1)	ARF Nucleotide Change (NM_058195.3)	p14 Protein change (NP_478102.2)	CM cases	MPM cases	Average age first melanoma	Pancreas cancer	Other cancer	Mutation published
Exon 1α	c.9_32del24	p.(A4_P11del)	None	None	2	0	45	0	0	[50]
Exon 1α	c.9_32del24	p.(A4_P11del)	None	None	2	1	64	0	0	[51]
Exon 1α	c.9_32dup24	p.(A4_p11dup)	None	None	2	0	54	0	Bladder	[9]
Exon 1α	c.9_32dup24	p.(A4_p11dup)	None	None	1	1	33	0	0	[9]
Exon 1α	c.47_50del	p.(L16Pfs*9)	None	None	1	1	40	1	0	New
Exon 1α	c.94_99dup	p.(L32_E33dup)	None	None	3	3	25	0	0	New
Exon 1α	c.103G>A	p.(G35R)	None	None	1	1	28	0	0	unpublished data
Exon 1α	c.103G>A	p.(G35R)	None	None	4	2	52	0	SCC, CLL	unpublished data
Exon 1β	None	None	c.62G>A	p.(R21K)	1	0	54	0	0	New
Intron 1	None	None	c.193+5G>A	Splice defect	9	4	38	0	Cervix	[45]
Intron 1	None	None	c.193+5G>A	Splice defect	4	2	45	0	RCC	[45]
Exon 2	c.301G>T	p.G101W	c.344G>T	p.R115L	3	2	57	0	Breast	[9]
Exon 2	c.335_337dup	p.A112dup	c.379_381dup	p.(S127dup)	7	4	34	0	Breast, Lung	[49]

SCC: squamous cell carcinoma

CLL: chronic lymphocytic leukemia

RCC: renal cell carcinoma

The novel frameshift mutation c.47_50del, p.(L16Pfs*9) is likely to be highly deleterious to the p16 protein function since it causes premature truncation of the protein. Furthermore the mRNA transcript may also be subject to nonsense mediated decay. This was present in one MPM case, with a father who died of pancreatic cancer (unavailable for genotyping).

In one family we found a duplication of 6 bp (c.94_99dup, p.(L32_E33dup)) causing a 2 amino acid duplication in the first ankyrin-repeat of p16. The mutation segregated in a 3-case CM family where all had MPM at young age.

A missense mutation in p14 (c.62G>A, p.(R21K), exon 1β) was identified in an individual affected with CM aged 54 years and no family history of CM. To-date, no melanoma families have been identified that carry missense mutations in exon 1β, however, very recent studies have shown that p14-specific alterations in CDKN2A exon 2 impair the ability of p14 to control superoxide levels and suppress growth of melanoma cells in vivo [43]. Previously, only whole gene deletions, insertions or splice-site mutations in p14, have been determined as pathogenetic [44].

In two non-related families we found a probable splice-site mutation (c.193+5G>A) which segregated with CM in both families. One family has previously been described [45] with 9 persons affected with CM, many with MPM, and segregation of the mutation with melanoma resulting in a LOD-score of 3.6. In the second non-related family the mutation segregated in a family with 4 CM cases, two of whom had MPM. The mutation has previously been described as a somatic mutation [46], and is located in a conserved area of intron 1. The mutation is located 5 nucleotides from the splice-donor site of p14ARF, and therefore affects only p14. The Human Splice Finder version 2.4.1 algorithm (http://www.umd.be/HSF/) predicted reduced splice signal strength, but the effect has not been examined at the mRNA level.

The missense mutation p.(G35R) in p16 has previously been found in melanoma cases (unpublished data) as well as in tumor tissue. *In silico* and functional prediction analysis graded the mutation as a class 3 mutation (Uncertain) [47] in the 5-class score system (IARC 5-class classification system). However, the mutation was observed in 2 of 327 examined high-risk melanoma patients from the current project but were absent from 1965 Danish control exomes from a diabetes study [48]. In one family the mutation was present in the two siblings with CM who were available for examination. Two other siblings with CM were deceased and genotyping was not possible. The other individual heterozygous for p.(G35R) mutation had MPM in young age, and no maternal history of cancer. The biological father was an anonymous sperm donor, and thus unavailable for follow up.

Several known mutations were also found in *CDKN2A*. One family of Swedish descent carried the known Swedish founder mutation: p.R112dup [49]. Additionally, we observed the p.(A4_P11dup), p.(A4_P11del), and p.G101W mutations [50] [9,51].

In 18 cases we found the well-described *CDKN2A* p.A148T variant. The minor allele frequency (MAF) of this polymorphism reported in the European American cohort of the ESP database is 0.0225, which correlates well with the frequency in our data set (0.0275).

The average age of first melanoma was 42.8 years in *CDKN2A* mutation carriers (excluding those carrying the missense variant in p14), which is significantly younger (48.3 years, p = 0.035) than non-*CDKN2A* mutation carriers (Table 2).

**Table 2:** Age at first melanoma in *CDKN2A* mutation carriers compared to age of first melanoma in individuals with melanoma and no *CDKN2A* mutation.

*CDKN2A* mutation	N	Mean age of first CM	Median age of first CM	Std Dev	Likelihood Ratio
0	571	48.3	50	15.4	Reference
1	34	42.8	42	13.7	0.0349

Families with a *BAP1* mutation are not included, nor are individuals with UM.

Overall, we analysed *CDKN2A* in 304 unrelated melanoma cases suspected of a hereditary predisposition to CM and found a pathogenetic mutation in 3.9% (Table 3). In 107 individuals with MPM or melanoma before 40 years, we found 3 *CDKN2A* mutations, all in individuals with MPM, first diagnosed with CM aged 28, 33, and 40 years, respectively. In two of these, knowledge of the paternal family history was limited or absent, and the father of the third individual died of pancreatic cancer aged 49 years. In families with 3 CM cases, we found mutations in 5.6%, and in families with 4 or more cases mutations were found in 23.5%. No *CDKN2A* mutations were found in 6 individuals with UM or 17 families with UM and CM. Similarly, no *CDKN2A* mutations were seen in 3 individuals with isolated pancreatic cancer, and 6 individuals with pancreatic cancer and a first-degree relative with pancreatic cancer. Among 15 families with pancreatic cancer and CM, we found 1 family with a *CDKN2A* mutation. In the 13 families with *CDKN2A* mutations, only 1 had a case of pancreatic cancer, in a person with unknown carrier status (the same family as above) (Table 1).

**Table 3:** *CDKN2A* analysis of individuals with CM.

	Total single affected	Single affected with one CM	Single affected with MPM	Two second degree relatives with CM	Two first degree relatives with CM	Both with single CM	One or two with MPM	Three affected	Four affected	Total	3+ (3 or more affected)
Examined	107	37	70	24	120	69	51	36	17	304	53
Mutations	3	0	3	0	3	2	1	2	4	12	6
%	2.8	0	4.3	0	2.5	2.9	2.0	5.6	23.5	3.9	11.3

**Fig 2:**
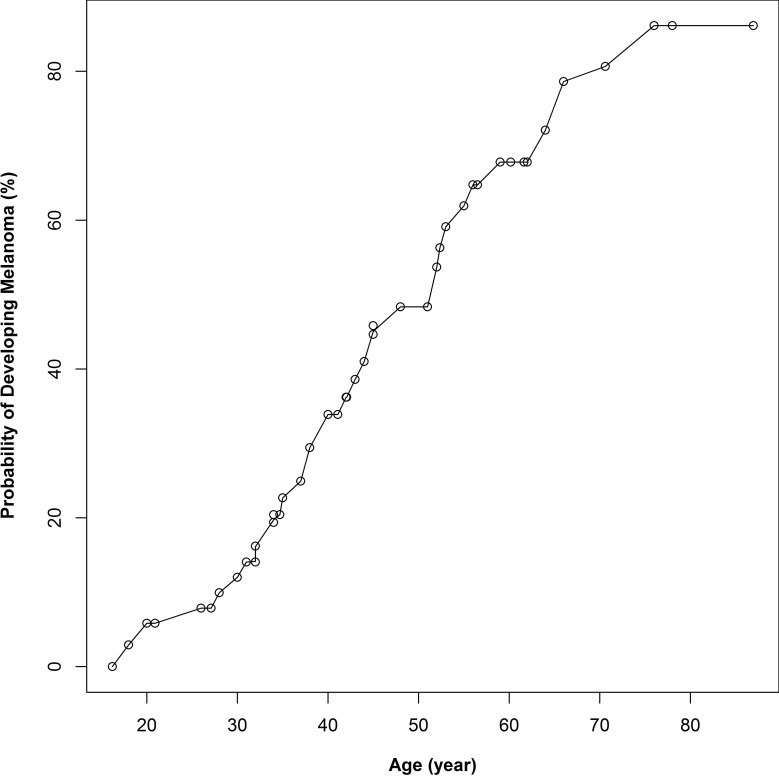
Age-specific penetrance curves for CM in Danish *CDKN2A* mutation carriers.

The calculated age-specific penetrances for CM in *CDKN2A* mutation carriers are shown in Fig. 2. The estimated penetrance at age 70 is 80%. In the 12 *CDKN2A* mutation positive families we identified 34 cases with CM, of which 27 (79%) were known gene carriers. 18 *CDKN2A* gene carriers were unaffected, and had a mean current age of 47 years.

### CDK4

Of the 327 families examined, a *CDK4* mutation (p.R24H) was only found in one. This family had 3 CM cases, two with MPM. The affected parent was adopted and had no information of the biological family. This illustrates that *CDK4* mutations are very rare in Denmark, which is in accordance with reports from other countries [17].

### BAP1

Using T-NGS the mean sequence coverage in 136 samples was 30X or above. The sequencing of these samples was considered to be of sufficient depth to give accurate mutation data regarding *BAP1*. We analysed *BAP1* in 10 cases of CM < 40 years, in 23 cases of MPM, and in 94 CM families, and found no *BAP1* mutation (Table 4). In 12 individuals with UM and unknown family history of cancer, we found no *BAP1* mutations. We analysed *BAP1* in 10 families with 1 case of UM and 1 or more cases of CM, and found no *BAP1* mutation. We analysed *BAP1* in 6 families with 2 or more cases of UM and found truncating *BAP1* mutations in 4 families (66.7%), all of which have been published [33,52,53]. We analysed 5 families with CM and mesothelioma, and found truncating *BAP1* mutations in 2 of the families. In 1 of these families there were also 3 cases of UM [53], but in the other there was no case of UM [33].

**Table 4:** *BAP1* analysed in individuals and families with CM and/or UM and mesothelioma.

Cancers	Examined	Mutations	%
Sporadic CM case with onset <40 years	10	0	0
Sporadic MPM case	23	0	0
Familial CM	94	0	0
Sporadic UM case	12	0	0
CM family with 1 UM case	10	0	0
Family with 2 UM cases +/- CM	6	4	66.7
CM family with mesothelioma case	5	2	40

### MC1R

In the CM cohort we observed a MAF of R variants of 0.389 (Table 5), which is almost twice the frequency in the Danish population [48]. In the Danish CM cases there is a particularly high frequency of p.R151C (MAF 0.173). We observed a MAF of 0.236 for the r variants in the cohort of CM cases, which is very close to the population MAF of 0.241.

**Table 5:** Minor allele frequency (MAF) and odds ratio (OR) of *MC1R* variants in melanoma cases/families compared to the Danish population.

Variant	Population (MAF)	Single person with CM	OR	Familial CM	OR	total	OR
No. of cases	1965	**45**		**235**		**280**	
**R (total)**	**0.204**	**0.46**	**2.24**	**0.40**	**1.97**	**0.389**	**1.913**
p.D84E	0.015	0.022	1.533	0.028	1.908	**0.027**	**1.847**
p.R142H	0.003	0.011	3.359	0.000		**0.002**	**0.540**
p.R151C	0.084	0.178	2.111	0.164	1.945	**0.166**	**1.972**
p.R160W	0.088	0.211	2.412	0.136	1.556	**0.148**	**1.693**
p.D294H	0.014	0.033	2.383	0.019	1.369	**0.021**	**1.532**
p.N29K-INS.A	0	0		0.013		**0.020**	
c.284C-T	0	0		0.002		**0.002**	
c.637_655del	0	0		0.004		**0.004**	
**r (total)**	**0.241**	**0.167**	**0.691**	**0.249**	**1.032**	**0.236**	**0.977**
p.V38M	0.002	0		0.002	1.393	**0.002**	**1.169**
p.V60L	0.101	0.056	0.553	0.102	1.016	**0.095**	**0.942**
p.V92M	0.075	0.078	1.036	0.083	1.105	**0.082**	**1.094**
p.A149T	0	0		0.002		**0.002**	
p.R163Q	0.053	0.033	0.627	0.055	1.040	**0.052**	**0.974**
Rare r	0.011	0		0		**0**	

*CDKN2A* mutation carriers with *MC1R* variants had a hazard ratio of 3.39 for developing CM compared to *CDKN2A* mutation carriers with no *MC1R* variants. *CDKN2A* mutation carriers with one or two R variants had a hazard ratio of 2.52, and *CDKN2A* mutation carriers with one or two r variants had a hazard ratio of 2.24 (Table 6). *CDKN2A* mutation carriers with [R/R, R/r] *MC1R* genotypes had a statistically significant (p = 0.038) increased OR (6.16) for developing CM compared to *CDKN2A* mutation carriers with [R/wt, r/r, r/wt, wt/wt] *MC1R* genotypes, and a statistically significant (p = 0.025) increased risk of developing melanoma 10 years earlier, with an OR of 2.25. We also found that *CDKN2A* mutation carriers with [R/R, R/r] *MC1R* genotypes, were 24 times more likely to have MPM compared to carriers with the [wt/wt] *MC1R* genotype (p = 0.033).

**Table 6:** Hazard ratios for CM in *CDKN2A* carriers according to *MC1R* genotype, compared to all *CDKN2A* carriers.

Risk factor	Hazard Ratio	95% CI	p-value
*MC1R* variant	3.39	0.75–15.25	P = 0.112
R variant	2.52	0.92–6.91	P = 0.0714
r variant	2.24	0.35–14.49	P = 0.396

Using Cox regression

### MITF

The *MITF* p.E318K mutation was analysed in DNA from 276 participants with CM, and we found 4 carriers (Table 7). None of 20 individuals with UM carried the mutation. In the Danish population [48] the MAF of *MITF* p.E318K was 9/3930 = 0.0023. We observed an OR of 3.16 in Danish melanoma patients.

**Table 7:** MAF and OR of *MITF* p.E318K in this study compared to the Danish population, and compared to other cohorts of CM patients.

Population & reference	Carriers	MAF	OR
Danish 1965 controls	9/1965	0.0023	reference
This study CM	4/276	0.0072	3.16
This study UM	0/20	0	
French CM [26]	17/603	0.014	4.78
Italian CM [62]	12/667	0.011	2.85
Australian CM [25]	34/2025	0.0165	2.33
UK CM [25]	34/1895	0.0176	2.09
Polish CM [63]	2/748	0.001	1.11

## Discussion

We identified *CDKN2A* mutations in 3.9% of unrelated high-risk Danish CM cases. The frequency of *CDKN2A* mutations in population based CM cases is 2% in North America, Europe and Australia [54], so a frequency of 3.9% in high-risk CM cases is surprisingly low. This is further illustrated by the fact that we only found *CDKN2A* mutations in 5.6% of 3-case CM families, where previous reports have found mutation in 30% and 40% of such families from North America and Europe, respectively [9]. However, in Australia, another high-risk country for CM like Denmark, only ∼10% of 3-case CM families carried a *CDKN2A* mutation [9].

We did not find any founder mutations in *CDKN2A* in Danish families, and only one family, of Swedish descent, was identified with the known Swedish founder mutation. As reported in other studies we found that carriers of *CDKN2A* mutations generally develop CM earlier, mean age 42.8 years, than other high-risk CM cases, mean age 48.3 years. The penetrance for *CDKN2A* mutation carriers was 50% at age 50 and 80% at age 70, which is in keeping with the previously observed penetrances in North America and Australia [51,55], but considerably higher than the penetrance observed in other European countries [51]. Interestingly, Bishop et al 2002 [51], had excluded Sweden from the European penetrance calculations because of an observed higher penetrance compared to other European countries. Previously, it has been shown that the penetrance of *CDKN2A* mutations is greater in a high-risk cohort, compared to cases identified through screening of an unselected sample of melanoma cases [54].

We found a low frequency of pancreatic cancer in *CDKN2A* mutation-positive families in Denmark, and only 1 of 15 families (6.7%) with pancreatic cancer and CM had a *CDKN2A* mutation, in a person with unknown carrier status. This is in contrast to other reports, where in North America and Europe *CDKN2A* mutations were observed in 70–80% of families with pancreatic caner and 3 cases of CM, and in Australia a *CDKN2A* mutation was only found in 30% of such families [9]. Only 3 of the 15 pancreatic cancer and CM families in this study had 3 cases of CM together with one case of pancreatic cancer, and in none of these families was a *CDKN2A* mutation identified. It is unknown if pancreatic cancer among *CDKN2A* mutations carriers in different geographic regions is caused by life-style factors, environmental factors, or genetic modulators. Alternatively, there may be a genotype-phenotype correlation between the position of mutations in *CDKN2A* and risk of pancreatic cancer. In families with pancreatic cancer only we did not find *CDKN2A* mutations, which is in contrast to observations in Dutch and Italian pancreatic cancer families [18,56], but in accordance with reports from North America and Germany [57,58]. Our results are not sufficient to exclude pancreatic cancer as part of the phenotype in Danish *CDKN2A* carriers and it is unknown if there is an increased risk of cancers other than CM.

In this study we examined *BAP1* in 133 high-risk CM cases (Table 4) and found no mutations, but identified mutations in 4/16 (25%) UM-CM families, all of which had 2 or more cases of UM (Table 4). This is in line with previous reports by Njauw et al, where they found *BAP1* mutations in 0.5% of CM families [59], and in 28.5% of UM-CM families. One of the weaknesses of the study is that we did not recruit UM patients in a systematic manner, however, we are in the process of examining 100 UM patients for germline *BAP1* mutations. We found *BAP1* mutations in 40% of families with CM and mesothelioma (Table 4). Thus, in the Danish population, it seems warranted to screen primarily only those families in which there are CM and UM and/or mesothelioma. We did not find *BAP1* mutations in 3 families with CM and RCC. Whether *BAP1* screening should be conducted in Danish families with CM and RCC remains unclear and further studies are needed to examine the frequency of *BAP1* mutations in families with RCC, with and without CM.

In Danish CM cases the frequency of *MC1R* R variants is high (39%, almost twice the frequency of controls, MAF 0.2). Several other studies from multiple countries have shown an OR between 2–3 for R variants in CM cases compared to the relevant control population [60]. The Danish population has a high frequency of R variants, almost double that of the French population [61], but only marginally higher than the Swedish population and lower than the Icelandic population [27]. The high frequency of R variants in combination with environmental UVR-exposure (either sunlight or indoor tanning) could account for some of the CM cases. In cohorts of CM cases from Southern Europe the OR for association with CM of *MC1R* r variants has been reported to be highly variable (between 0.84–3) [60]. In Northern Europe the OR for association with CM has been consistently low (between 0.58–1.31) [60], which is in concordance with the observed OR in the Danish sample reported here (0.977).

As Denmark is a high incidence country for melanoma [1], there is a distinct possibility of phenocopies in families, and since only one person from each family was examined for *CDKN2A* and *CDK4* mutations, it cannot be ruled out that mutations in some families have not been identified. Alternatively, mutations in other yet unknown predisposition genes could explain the low rate of *CDKN2A* mutations identified. To address this possibility we are in the process of exome sequencing melanoma families with 3 or more CM cases without identified mutations in *CDKN2A* or *CDK4*. Such an approach has previously identified new genes and pathways relevant to melanoma susceptibility [13,14]. Mutations in *CDK4*, and the *MITF* p.E318K mutation, are rare in the Danish population examined here, and only explain a minority of CM cases. The MAF of *MITF* p.E318K in Danish CM cases (0.0072) is lower than previously observed in UK CM cases (0.0176), Australian CM cases (0.0165)[25], and Italian and French CM cases (MAF 0.011 and 0.014)[26,62], respectively. However, it is higher than the MAF observed in Polish CM cases (0.001)[63]. The OR in Danish CM cases of 3.16 is in the middle of previously observed ORs (Table 7), where the outliers are France, with an OR of 4.78, and Poland, with an OR of 1.11.

*MC1R* is a modulator of *CDKN2A* mutations and we found a trend of carrying any *MC1R* variant being associated with increased risk of CM in *CDKN2A* mutation carriers (Table 6). Although these findings are not significant, probably due to small sample size, the trend and point estimates are in keeping with prior observations in a different population [55]. *CDKN2A* mutation carriers with [R/R, R/r] *MC1R* genotypes, had a significantly higher risk of developing melanoma compared to other carriers, and had an OR of 2.25 for developing CM 10 years earlier than carriers with [r/r, R/wt, r/wt, wt/wt] *MC1R* genotypes. It has previously been shown that *MC1R* variants increased the risk of melanoma in *CDKN2A* mutation carriers [55,64], however in Italian *CDKN2A* mutations carriers, who have few *MC1R* variants, other factors influence the risk of developing CM [65]. We found that *CDKN2A* mutation carriers with (R/R, R/r) *MC1R* genotypes had significantly higher risk of developing MPM compared to carriers with wt *MC1R* genotype.

To conclude, we propose that mutation screening of *CDKN2A* and *CDK4* in Denmark should predominantly be performed in families with at least 3 cases of CM, and in individuals with MPM. The latter should be screened predominately when family history of CM or pancreatic cancer is unknown, since we identified three *CDKN2A* mutations in individuals with MPM and all had no or limited information about their family cancer history. The age-specific penetrance for CM in *CDKN2A* mutation carriers is high in Denmark, as in other high incidence melanoma countries [51], and *MC1R* variants modulate the penetrance of CM and the risk of MPM. However, other factors, for instance UVR-exposure, might influence the penetrance of CM in *CDKN2A* carriers, and in a clinical setting, analysis of *MC1R* in mutation carriers is not indicated, as it would be unlikely to alter the surveillance program or recommendation of taking sun-protective precautions. The *MITF* p.E318K mutation is a rare moderate risk CM allele in the Danish population. At present, routine clinical testing of *MITF* p.E318K in CM patients does not appear warranted. The occurrence of UM in a family with CM points toward other genes than *CDKN2A* as a causative component, thus screening for *CDKN2A* mutations in such families does not seem warranted. We recommend that testing of *BAP1* should not be conducted routinely in CM families but reserved for families with CM and UM, or mesothelioma (Table 8), and possibly also families with the occurrence of RCC [66]. Finally, genetic testing should always be conducted as part of genetic counselling, and since CM can be part of a variety of cancer syndromes, validation of cancer diagnosis [67] in a family is crucial for correct counselling.

**Table 8:** Recommendations for genetic testing in Danish melanoma cases/families, conducted as part of genetic counselling.

	*CDKN2A / CDK4*	*BAP1*
Genetic testing should be offered	• Families with 3 or more affected with CM	• Families with 2 or more cases of UM and/or mesothelioma
		• Individual with UM and mesothelioma
Genetic testing should be considered	• Individual with MPM, and sparse family history	• Families or individuals with any combination of two or more of these cancers: CM, RCC, UM, mesothelioma
	• Two first degree relatives affected with CM, and sparse family history	

## Supporting Information

S1 Dataset (DOCX)
