# Supplementary material for: Molecular Characterization of Melanoma Cases in Denmark Suspected of Genetic Predisposition
Source: PLoS One. 2015 Mar 24;10(3):e0122662. doi: 10.1371/journal.pone.0122662 (PMC4372390; doi:10.1371/journal.pone.0122662)
Supplement: S1 Dataset — (DOCX) [file pone.0122662.s001.docx]

S1 Dataset

Primers (all written 5’ to 3’):

*CDKN2A*:

*CDKN2Ae*x1aFH acccactgcttactggcttatcACCGGAGGAAGAAAGAGGAG

*CDKN2A*ex1aRH gaggggcaaacaacagatggcAACCCCTTCTGAAAACTCCC

CDKN2Aex2FH acccactgcttactggcttatcACTGGAAGCAAATGTAGGGG

CDKN2Aex2RH gaggggcaaacaacagatggcTGAGGCAAGACCGGAGAC

CDKN2Aex1bFH acccactgcttactggcttatcCTCAGGGAAGGCGGGTG

CDKN2Aex1bRH gaggggcaaacaacagatggcAGTCGTTGTAACCCGAATGG

CDKN2Aex3FH_2 acccactgcttactggcttatcGTGAAGCCATTGCGAGAACT

CDKN2Aex3RH_2 gaggggcaaacaacagatggcCGTTAAAAGGCAGGACATTTTT

*CDK4*:

CDK4ex2F tgtaaaacgacggccagtTGGGAGTCCCTTTGTTGCTGCAGG

CDK4ex2R caggaaacagctatgaccGGTGTGATGATCTGTAGAGAAGTG

*MITF:*

MITF_E148K_F TGCTCTCCAGATTTGGTGAATCG

MITF_E148K_R GGTCTTGGCTGCAGTTCTCAA

*MC1R*:

MC1R_F gcagcaccatgaactaagcag
MC1R_R ccagaccacacaatatcaccac

*BAP1:*

Primers used from [12] Wiesner T, Obenauf AC, Murali R, Fried I, Griewank KG, Ulz P, et al. Germline mutations in BAP1 predispose to melanocytic tumors. Nat Genet 2011 Oct;43(10):1018-21. ([http://www.ncbi.nlm.nih.gov/pubmed/21874003?dopt=Citation](https://mail/owa/redir.aspx?C=VEprRZQef0-EXhdpCyunuBIZCKBo19EI9Ei_YG-alnzArA-3D3I_9zFLYgZZ3edbHjTYxblhQ1s.&URL=http%3a%2f%2fwww.ncbi.nlm.nih.gov%2fpubmed%2f21874003%3fdopt%3dCitation))
